# Performance Enhancement for a GPS Vector-Tracking Loop Utilizing an Adaptive Iterated Extended Kalman Filter

**DOI:** 10.3390/s141223630

**Published:** 2014-12-09

**Authors:** Xiyuan Chen, Xiying Wang, Yuan Xu

**Affiliations:** 1 School of Instrument Science and Engineering, Southeast University, Nanjing 210096, China; E-Mail: wangxiying@outlook.com; 2 Key Laboratory of Micro-Inertial Instrument and Advanced Navigation Technology Ministry of Education, Southeast University, Nanjing 210096, China; 3 School of Automation and Electrical Engineering, University of Jinan, Jinan 250022, China; E-Mail: xy_abric@126.com

**Keywords:** Global Positioning System (GPS), iterated extended Kalman filter (IEKF), model error, nonlinear filtering, vector-tracking

## Abstract

This paper deals with the problem of state estimation for the vector-tracking loop of a software-defined Global Positioning System (GPS) receiver. For a nonlinear system that has the model error and white Gaussian noise, a noise statistics estimator is used to estimate the model error, and based on this, a modified iterated extended Kalman filter (IEKF) named adaptive iterated Kalman filter (AIEKF) is proposed. A vector-tracking GPS receiver utilizing AIEKF is implemented to evaluate the performance of the proposed method. Through road tests, it is shown that the proposed method has an obvious accuracy advantage over the IEKF and Adaptive Extended Kalman filter (AEKF) in position determination. The results show that the proposed method is effective to reduce the root-mean-square error (RMSE) of position (including longitude, latitude and altitude). Comparing with EKF, the position RMSE values of AIEKF are reduced by about 45.1%, 40.9% and 54.6% in the east, north and up directions, respectively. Comparing with IEKF, the position RMSE values of AIEKF are reduced by about 25.7%, 19.3% and 35.7% in the east, north and up directions, respectively. Compared with AEKF, the position RMSE values of AIEKF are reduced by about 21.6%, 15.5% and 30.7% in the east, north and up directions, respectively.

## 1. Introduction

Traditional Global Positioning System (GPS) receivers utilize scalar tracking loops (STL) to track the received GPS signals. All scalar tracking loops are independent of each other and ignore the internal relationship of each satellite [1]. When the received GPS signals degrade, the tracking loop inside the receiver may fail, therefore, reliable tracking loop operation is required to improve the tracking performance of GPS receivers.

Recently, the vector-tracking loop (VTL) consisting of a vector delay lock loop (VDLL) and a vector frequency lock loop (VFLL) was proposed for GPS receivers at low C/N0 ratios condition. For instance, Kim *et al.*, proposed an adaptive VTL for low-quality GPS signals [1]; Zhao *et al.*, proposed implementation and performance assessment of a vector-tracking method based on a software GPS receiver in [2]; Jafarnia-Jahromi proposed a detection and mitigation of spoofing attacks on a vector-based tracking GPS receiver [3]. The VTL was first proposed by Spilker [4]; it is an appealing and advanced structure which is able to provide an improved performance over traditional scalar lock loops. Depending on the correlation of each satellite signal, the VTL technique processes received satellite signals and user dynamics together rather than separately. Thus, a VTL GPS receiver can get sufficient total signal power to track the signal even if the signal quality from individual satellites is low [5–8].

In a VTL GPS receiver, the VTL navigation filter used to control the numerically controlled oscillators (NCO) in each satellite channel can aid all the tracking loops to track weak received signals with other satellite channels. Thus, as the core of the VTL navigation filter, the nonlinear Kalman filter should be carefully designed. In the field of the estimation for the nonlinear system, extended Kalman filter is one of the most common examples [3,5]. However, although the EKF has the advantage in real-time estimation, the linearization of a nonlinear system by Taylor series expansion, neglecting of the truncated high-order terms will introduce a truncated error, it is a biased estimator [9–11]. In order to overcome this problem, unscented Kalman filter (UKF) [12] and iterated EKF (IEKF) [13] are proposed. The UKF employs a deterministic sampling technique known as the unscented transform to pick a minimal set of sample points (so called sigma points) around the mean. These sigma points are then propagated through the non-linear functions, from which the mean and covariance of the estimate are then recovered [14]. However, the UKF needs to compute large numbers of samples. In order to overcome this problem, the IEKF is proposed to reduce the bias and the estimation error by increasing only a few simple iterative operations [9–11]. It should be point out that, the model error statistics in the UKF and IEKF are still prior estimates [9], while the noise is unknown in practice.

Hence, motivated by the problems mentioned above, new methods need to be studied. In this work, the AIEKF which combines the advantages of the Adaptive EKF (AEKF) [9] and the IEKF is proposed for the VTL GPS receiver. The remainder of this paper is organized in four sections: Section 2 introduces the IEKF, AEKF and AIEKF. Section 3 presents the AIEKF method applied to a GPS vector-tracking loop in detail. In Section 4, the performance of the proposed filter is illustrated by a road test and compared with that of the AEKF and IEKF in estimation accuracy. Finally, the conclusions are given.

## 2. Adaptive Iterated Extended Kalman Filter

In this section, a brief introduction to the IEKF is given. Furthermore, a modified IEKF named AIEKF is proposed.

### 2.1. Iterated Extended Kalman Filter

For a discrete-time nonlinear system, its model can be given by the following equations:

(1) $${\text{X}}_{k}=f({\text{X}}_{k-1})+\Gamma_{k-1}{\text{W}}_{k-1}$$

(2) $${\text{Z}}_{k}=h({\text{X}}_{k})+{\text{V}}_{k}$$

where **X***_k_* is the state vector at step *k, f*(**X***_k_*) is the nonlinear system function, **Γ***_k_* is the process noise driving matrix, **Z***_k_* is the observation vector, *h*(**X***_k_*)Is the nonlinear observation model, **W***_k_* is the process noise which is assumed to be drawn from zero mean Gaussian white noise with covariance **Q***_k_*, and **V***_k_* is the observation noise which is assumed to be zero mean Gaussian white noise with covariance **R***_k_*.

The IEKF used in this paper involves the following recursive equations [9,10]:

(3) $${\hat{\text{X}}}_{k/k-1}=\Phi_{k-1}{\hat{\text{X}}}_{k-1/k-1}$$

(4) $${\text{P}}_{k/k-1}=\Phi_{k-1}{\text{P}}_{k-1}\Phi_{k-1}^{T}+{\text{Q}}_{k-1}$$

where **X̂***_k_*_/_*_k_*_−__1_ is the priori state estimate at step *k*, $\Phi_{k}=\frac{\partial f\left({\hat{\text{X}}}_{k/k}\right)}{\partial {\hat{\text{X}}}_{k/k}}$ is the Jacobian matrix of *f*(**X̂***_k/k_***), P***_k_*_/_*_k_*_−__1_ is the priori error covariance matrix.

In addition to the standard EKF functions, a few iterative operations are involved in IEKF to reduce the bias and estimation errors of **X̂***_k/k_*_−__1_ and **P***_k_*_/_*_k_*_−__1_ given from Equations (3) and (4). The recursive steps are:

*Step 1:* Initialize ${\hat{\text{X}}}_{k/k}^{n}$ and ${\text{P}}_{k/k}^{n}$
  (5) $${\hat{\text{X}}}_{k/k}^{1}={\hat{\text{X}}}_{k/k-1}$$
  (6) $${\text{P}}_{k/k}^{1}={\text{P}}_{k/k-1}$$
  where *n* = 1, 2, 3…,*N* is the number of the iteration.
*Step 2:* Iterative processing
  (7) $${\text{K}}_{k}^{n}={\text{P}}_{k/k-1}{\text{H}}^{n}{\left({\hat{\text{X}}}_{k/k}^{n}\right)}^{T}{\left[{\text{H}}^{n}\left({\hat{\text{X}}}_{k/k}^{n}\right){\text{P}}_{k/k-1}{\text{H}}^{n}{\left({\hat{\text{X}}}_{k/k}^{n}\right)}^{T}+{\text{R}}_{k}\right]}^{-1}$$
  (8) $${\hat{\text{X}}}_{k/k}^{n+1}={\hat{\text{X}}}_{k/k}^{n}+{\text{K}}_{k}^{n}\left[{\text{Z}}_{k}-h^{n}\left({\hat{\text{X}}}_{k/k}^{n}\right)-{\text{H}}^{n}\left({\hat{\text{X}}}_{k/k}^{n}\right)\cdot \left({\hat{\text{X}}}_{k/k-1}-{\hat{\text{X}}}_{k/k}^{n}\right)\right]$$
  (9) $${\text{P}}_{k/k}^{n+1}=\left[\text{I}-{\text{K}}_{k}^{n}{\text{H}}^{n}\left({\hat{\text{X}}}_{k/k}^{n}\right)\right]{\text{P}}_{k/k}^{n}$$
  where $K_{k}^{n}$ is the filter gain, ${\text{H}}^{n}\left({\hat{\text{X}}}_{k/k}^{n}\right)=\frac{\partial h\left({\hat{\text{X}}}_{k/k}^{n}\right)}{\partial {\hat{\text{X}}}_{k/k}^{n}}$ is the Jacobian matrix, *n* is the number of iteration steps.
*Step 3:* Iteration finished
  (10) $${\hat{\text{X}}}_{k/k}={\hat{\text{X}}}_{k/k}^{N}$$
  (11) $${\text{P}}_{k/k}={\text{P}}_{k/k}^{N}$$

### 2.2. Adaptive Iterated Extended Kalman Filter

In the EKF and IEKF algorithms, the nonlinear system function and nonlinear measurement function are linearized by Taylor series expansion. Hence, the ignoring of higher order terms may introduce a truncation error. It is evident that both the Q and R for EKF and those for IEKF are prior estimates. There are uncertainties in the noise description, and the assumptions on the statistics of disturbances are violated since the availability of a precisely known model is unrealistic in practical situations. In order to overcome these problems, the noise statistics estimator is employed in the IEKF. For a discrete-time nonlinear system described in Equations (3) and (4), the adaptive iterated extended Kalman filter (AIEKF) algorithm utilizes a set of equations as follows:

(12) $${\hat{\text{X}}}_{k/k}^{1}={\hat{\text{X}}}_{k/k-1}$$

(13) $${\text{P}}_{k/k}^{1}={\text{P}}_{k/k-1}$$

(14) $${\text{K}}_{k}^{n}={\text{P}}_{k/k-1}{\text{H}}^{n}{\left({\hat{\text{X}}}_{k/k}^{n}\right)}^{T}{\left[{\text{H}}^{n}\left({\hat{\text{X}}}_{k/k}^{n}\right){\text{P}}_{k/k-1}{\text{H}}^{n}{\left({\hat{\text{X}}}_{k/k}^{n}\right)}^{T}+{\hat{\text{R}}}_{k-1}^{n}\right]}^{-1}$$

(15) $${\hat{\text{X}}}_{k/k}^{n+1}={\hat{\text{X}}}_{k/k}^{n}+{\text{K}}_{k}^{n}\left[{\text{Z}}_{k}-h^{n}\left({\hat{\text{X}}}_{k/k}^{n}\right)-{\text{H}}^{n}\left({\hat{\text{X}}}_{k/k}^{n}\right)\cdot \left({\hat{\text{X}}}_{k/k-1}-{\hat{\text{X}}}_{k/k}^{n}\right)\right]$$

(16) $${\text{P}}_{k/k}^{n+1}=\left[\text{I}-{\text{K}}_{k}^{n}{\text{H}}^{n}\left({\hat{\text{X}}}_{k/k}^{n}\right)\right]{\text{P}}_{k/k}^{n}$$

(17) $${\hat{\text{X}}}_{k/k}={\hat{\text{X}}}_{k/k}^{N}$$

(18) $${\text{P}}_{k/k}={\text{P}}_{k/k}^{N}$$

(19) $${\text{R}}_{k}={\hat{\text{R}}}_{k}^{N}$$

where ${\hat{\text{R}}}_{k}^{n}$ is the estimate of ${\text{R}}_{k}^{n}$, it is estimated by the time-varying noise statistics estimators with the following equations:

(20) $${\hat{\text{R}}}_{k}^{n}=(1-d_{k-1}){\hat{\text{R}}}_{k-1}^{n}+d_{k-1}\left(\begin{array}{l} \left[\text{I}-{\text{H}}_{k}^{n}\left({\hat{\text{X}}}_{k/k}^{n}\right)\right]{\text{V}}_{k}{\text{V}}_{k}^{T}{\left[\text{I}-{\text{H}}_{k}^{n}\left({\hat{\text{X}}}_{k/k}^{n}\right)\right]}^{T}+\\ {\text{H}}_{k}^{n}\left({\hat{\text{X}}}_{k/k}^{n}\right){\text{P}}_{k/k}^{n}{\left[{\text{H}}_{k}^{n}\left({\hat{\text{X}}}_{k/k}^{n}\right)\right]}^{T} \end{array}\right)$$

where *d_k_*_−1_ = (1−*b*)/(1−*b^k^*),0 < *b* < 1.

## 3. Adaptive Iterated Extend Kalman Filter to Vector-Tracking GPS Receiver

### 3.1. The Architecture of a Vector-Tracking Loop

The term Vector-Tracking Loop was first proposed by Spilker [15]. He presented a vector delay-lock loop (VDLL) algorithm, combining all the tracking channels and navigation functions. A performance analysis about VTL was well described by Benson [16] in 2007. His study showed that the EKF-based VTL has potential advantages to improve the noise performance and reacquisition ability of a tracking loop. As software receivers developed, many studies on VTL were conducted [17]. Several VTL implementation methods in software receivers and their field test results showing improved tracking performance were reported in [18,19].

**Figure 1.**
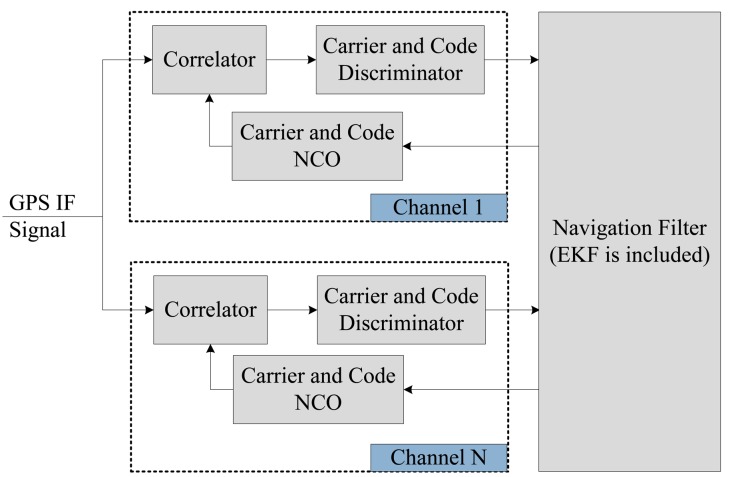
The architecture of the vector tracking loop.

Figure 1 shows the VTL architecture in the Global Navigation Satellite System (GNSS) receiver. Comparing with a conventional scalar tracking loop, a VTL tracking loop based on discriminator consists of a correlator, a discriminator and a code/carrier generator. The loop filter is removed in each channel. The discriminator outputs of each channel are directly connected to the navigation filter [20]. These are used as the measurement of the EKF in navigation filter.

The Doppler frequency and the pseudo range are calculated from the estimated user position and velocity of the navigation filter. Thus, there is one big loop including tracking channels and navigation module. Because the navigation result is derived from all channel tracking results, all the channels and navigation function are combined. This structure can track temporarily attenuated or blocked satellite signals because the navigation result can be derived from other visible satellites. In general, it is known that the vector-tracking loop based on the discriminator gives users a more accurate position and Doppler frequency than the scalar tracking loop. There have been many studies of VTL implementation. Recently, So [20] described VDLL, VFLL and VDFLL with EKF. As mentioned above, it can be seen that the filtering output accuracy of VTL is dependent on the EKF; however, the EKF will generate truncation errors due to Taylor's series expansion to linearize the nonlinear system. The VTL implementation in this paper is different from the previous studies. We implemented VTL with AIEKF in a VTL GPS software receiver.

### 3.2. Design of the AIEKF

**Figure 2.**
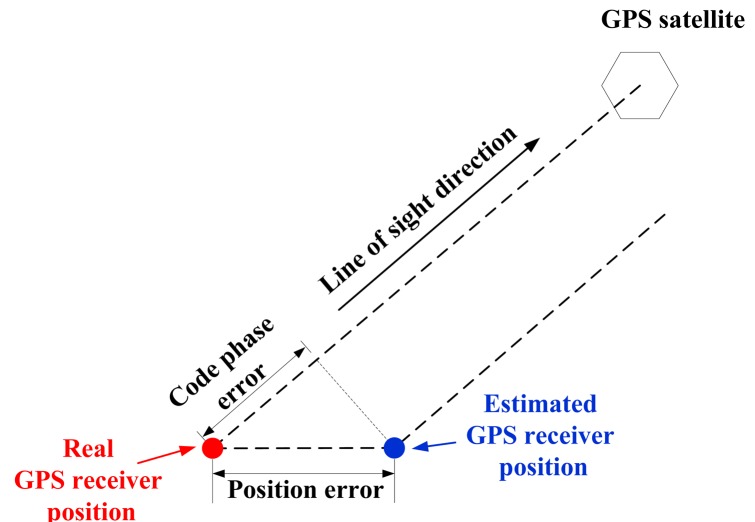
Relationship between GPS receiver position error and code phase error.

In a conventional GPS receiver, all channels process incoming signals independently. This architecture is easy to implement and channels do not affect each other if one of them loses lock. However, this independency also prevents one channel from helping another because information obtained from one is not utilized by others. Since all channels share the same receiver position and velocity, and feedbacks of the position and velocity from the navigation filter should be exploited by all tracking channels so that they can comprehensively process signals from different satellites. A GPS receiver's position error and velocity error are determined by the code phase errors and pseudo range error through a line-of-sight (LOS) projection, as shown in Figure 2. Ignoring all other non-Gaussian error sources such as satellite clock, multipath, hardware bias, *etc.*, the relationship between position error and code phase error can be written as the equation below [2]:

(21) $$\begin{array}{ll} \Delta \tau_{j,k} & =\tau_{j,k}-{\hat{\tau }}_{j,k}+w_{j,k}\\ & =t_{b,k}+{\left({\text{X}}_{k}-{\hat{\text{X}}}_{k}\right)}^{T}{\text{a}}_{j,k}+w_{j,k}^{\text{code}}\\ & =t_{b,k}+\Delta {\text{X}}_{k}\cdot {\text{a}}_{j,k}+w_{j,k}^{\text{code}} \end{array}$$

where the subscript *j* is the satellite number, the subscript *k* refers to measurement epoch, Δτ*_j,k_* is the code phase error in meters, τ*_j,k_* is the code phase measurement in meters, the symbol “^” represents the estimation of a variable, *t_b,k_* is the receiver clock bias in meters, **X***_k_* is the receiver position vector, ***a****_j,k_* is the unit LOS vector from the receiver to the jth satellite, and $w_{j,k}^{\text{code}}$ is the white Gaussian noise. Similarly, the carrier frequency error impacts the receiver velocity measurement error:

(22) $$\begin{array}{ll} \Delta f_{j,k} & =f_{j,k}-{\hat{f}}_{j,k}+w_{j,k}^{\text{carrier}}\\ & =t_{d,k}+{\left({\text{V}}_{k}-{\hat{\text{V}}}_{k}\right)}^{T}{\text{a}}_{j,k}+w_{j,k}^{\text{carrier}}\\ & =t_{d,k}+\Delta {\text{V}}_{k}\cdot {\text{a}}_{j,k}+w_{j,k}^{\text{carrier}} \end{array}$$

where Δ*f_j,k_* is the carrier frequency error, *f_j,k_* is the carrier frequency measurement, the symbol “^” represents the estimation of a variable, *t_d,k_* is the receiver clock drift in meters per second, **V***_k_* is the GPS receiver velocity vector, and $w_{j,k}^{\text{carrier}}$ is the white Gaussian noise. In a typical GPS receiver, the code phase and carrier frequency measurements in Equations (21) and (22) can be obtained from the tracking loops. The following sections show how to use these measurements so that a vector-tracking loop can be formed.

To obtain the dynamic equation for the local filter, the signal dynamic models are derived as follows. The user dynamics can be modeled using the shaping filter driven by white noise. When the user is stationary or moving with nearly constant velocity, an adequate model for the LOS range dynamic would be the integrated random walk. The discrete time state model for this integrated random walk model is:

(23) $${\text{X}}_{k+1}={\text{X}}_{k}+\text{T}\cdot {\text{V}}_{k}$$

where T is the discrete time interval.

The acceleration of the receiver is modeled as a Gaussian distribution white noise, and thus the velocity at epoch *k* + 1 is expressed as below:

(24) $${\text{V}}_{k+1}={\text{V}}_{k}+v_{k}$$

The drift of the receiver clock is assumed a constant plus a small white noise. So the clock bias and drift are modeled by Equations (25) and (26):

(25) $$t_{b,k+1}=t_{b,k}+\text{T}\cdot t_{d,k}$$

(26) $$t_{d,k+1}=t_{d,k+1}+\eta_{k}$$

where *t_b,k_* is the GPS receiver clock bias at step *k, t_d,k_* is the GPS receiver clock drift at step *k*, η*_k_* is the GPS receiver clock drift noise.

#### 3.2.1. Process Equation of Vector-Tracking Loop

The process equation can be established based on Equations (23)–(26). The receiver position error, velocity error, clock bias, and clock drift are the state variables. Equation (27) shows the discrete process equation:

(27) $$\left[\begin{array}{c} \Delta {\text{X}}_{k+1}\\ \Delta {\text{V}}_{k+1}\\ t_{b,k+1}\\ t_{d,k+1} \end{array}\right]=\Phi_{k,k+1}\left[\begin{array}{c} \Delta {\text{X}}_{k}\\ \Delta {\text{V}}_{k}\\ t_{b,k}\\ t_{d,k} \end{array}\right]+{\text{W}}_{k}$$

(28) $$\Delta {\text{X}}_{k}={\left[\begin{array}{ccc} x_{k}-{\hat{x}}_{k} & y_{k}-{\hat{y}}_{k} & z_{k}-{\hat{z}}_{k} \end{array}\right]}^{T}$$

(29) $$\Delta {\text{V}}_{k}={\left[\begin{array}{ccc} v_{x,k}-{\hat{v}}_{x,k} & v_{y,k}-{\hat{v}}_{y,k} & v_{z,k}-{\hat{v}}_{z,k} \end{array}\right]}^{T}$$

(30) $$\Phi_{k,k+1}=\left[\begin{array}{llllllll} 1 & 0 & 0 & \text{T} & 0 & 0 & 0 & 0\\ 0 & 1 & 0 & 0 & \text{T} & 0 & 0 & 0\\ 0 & 0 & 1 & 0 & 0 & \text{T} & 0 & 0\\ 0 & 0 & 0 & 1 & 0 & 0 & 0 & 0\\ 0 & 0 & 0 & 0 & 1 & 0 & 0 & 0\\ 0 & 0 & 0 & 0 & 0 & 1 & 0 & 0\\ 0 & 0 & 0 & 0 & 0 & 0 & 1 & \text{T}\\ 0 & 0 & 0 & 0 & 0 & 0 & 0 & 1 \end{array}\right]$$

where Δ**X***_k_* is the GPS receiver position error at step *k*, Δ**V***_k_* is the GPS receiver velocity error at step *k*, **Φ***_k_*_,_*_k_*_+1_ is the state transition matrix.

#### 3.2.2. Measurement Equation of Vector-Tracking Loop

The code phase discriminator and carrier frequency discriminator provide noisy code phase error and carrier frequency error when working within their linear range [17]. As shown in Equations (23) and (24), the receiver position and velocity are directly affected by the code phases and carrier frequencies observables. Therefore, the outputs of the discriminators are used as the measurements for the AIEKF as shown in Equation (32):

(31) $${\text{Z}}_{k}=h(\delta {\text{X}}_{k})+{\text{V}}_{k}$$

(32) $${\text{Z}}_{k}={\left[\begin{array}{cccc} E_{\text{code},1,k} & \ldots & E_{\text{code},J,k} & \begin{array}{cc} E_{\text{carrier},1,k} & \begin{array}{cc} \ldots & E_{\text{carrier},J,k} \end{array} \end{array} \end{array}\right]}^{T}$$

(33) $$\delta {\text{X}}_{k}={\left[\begin{array}{cccc} \Delta {\text{X}}_{k} & \Delta {\text{X}}_{k} & t_{b,k} & t_{d,k} \end{array}\right]}^{T}$$

where **Z***_k_* is the measurements, *E_code,j,k_* is the code phase discriminator output of channel *j* (0 ≤ *j* ≤ *J*), *E_carrier,j,k_* is the carrier frequency discriminator output of channel j (0 ≤ *j* ≤ *J*), δ**X***_k_* is the state vector, *h*(·) is the nonlinear observation equation, **V***_k_* is Gaussian distribution white noise.

When Equation (31) is used in AIEKF, it can be linearized by Taylor series expansion and expressed as in Equation (34).

(34) $${\text{Z}}_{k}=\text{H}\delta {\text{X}}_{k}+{\text{V}}_{k}$$

(35) $$\text{H}=\left[\begin{array}{cccccccc} a_{x,1} & a_{y,1} & a_{z,1} & 0 & 0 & 0 & -1 & 0\\ \vdots & \vdots & \vdots & \vdots & \vdots & \vdots & \vdots & \vdots \\ a_{x,J} & a_{y,J} & a_{z,J} & 0 & 0 & 0 & -1 & 0\\ 0 & 0 & 0 & a_{x,1} & a_{y,1} & a_{z,1} & 0 & -1\\ \vdots & \vdots & \vdots & \vdots & \vdots & \vdots & \vdots & \vdots \\ 0 & 0 & 0 & a_{x,J} & a_{y,J} & a_{z,J} & 0 & -1 \end{array}\right]$$

where $\text{H}=\frac{\partial h\left(\delta {\text{X}}_{k}\right)}{\partial \delta {\text{X}}_{k}}$ is the Jacobian matrix, *a_x,j_, a_y,j_* and *a_z,j_* are the components of the LOS vector pointing from the receiver to the number j satellite.

#### 3.2.3. Implementation of AIEKF to Vector-Tracking Loop

**Figure 3.**
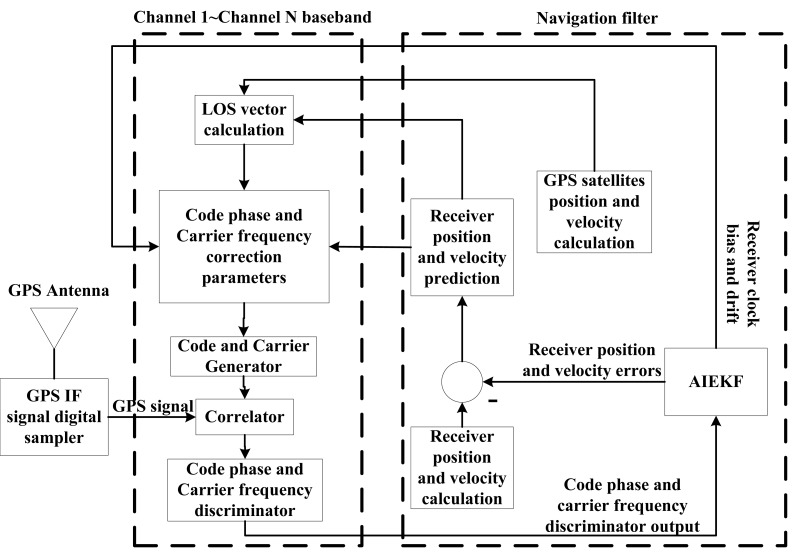
Flowchart of vector-tracking loop based on AIEKF.

**Figure 4.**
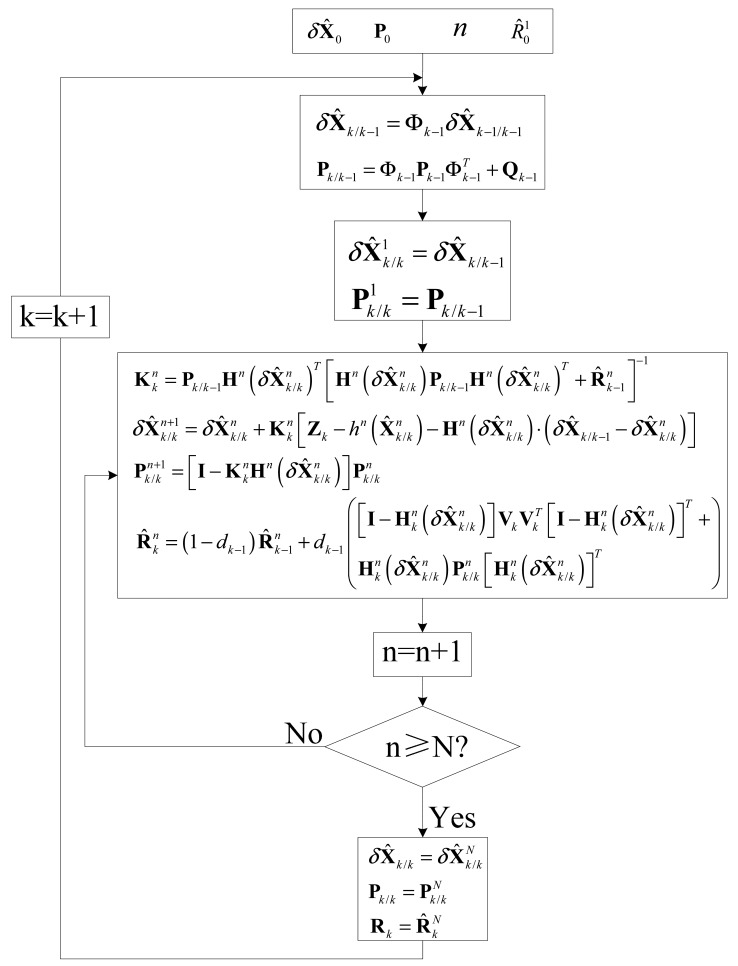
Flowchart for implementing AIEKF.

As mentioned in the previous section, the GPS receiver position error, velocity error, receiver clock bias and clock drift terms consist of the AIEKF state vector, while the code phase and carrier frequency discriminator outputs are the measurement of AIEKF. The block diagram for GPS software defined VTL receiver using AIEKF is shown in Figure 3. Figure 4 illustrates the flow chart for implementing the proposed AIEKF.

#### 3.2.4. Effectiveness of IEKF in VTL

**Figure 5.**
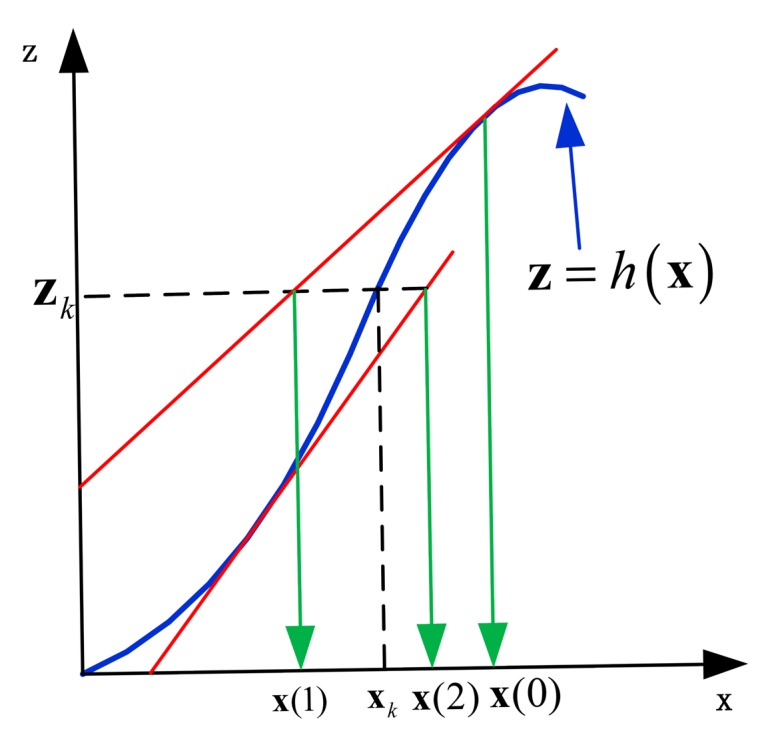
Graphic fundamental of IEKF with two iterations.

Shojaie *et al.* [21] uncovered the fact that the iteration of the observation updating step in a Kalman filter family will reduce the linearization error and improve estimation accuracy. The detailed ideas are explained with IEKF. The observation function *h*(**X***_k_*) is expanded with Taylor series at **x̂***_k,k_*_−1_, which is **x**(0) in Figure 5. The linear expansion of the observation function can be described as:

(36) $${\text{Z}}_{\text{lin}}=h\left({\hat{\text{X}}}_{k,k-1}\right)+\nabla_{x}h({\text{X}}_{k}-{\hat{\text{X}}}_{k,k-1})$$

If the real observation is **z***_k_*, the state estimate **x**(1) at time k can be obtained with Equation (36) and the Equation **z***_lin_* **= z***_k_*.

It can be seen from Figure 5 that there is an obvious distance between **x**(1) and **x***_k_*. This distance comes from the truncation error brought by the linearization of *h*(**X***_k_*), which is expanded with Taylor series. Shojajie found that the re-linearization of the observation equation can reduce the error between estimated value and observed value. The iterated extended Kalman filter repeatedly calculates the Kalman gain and an intermediate posterior state estimate ${\hat{\text{X}}}_{k}^{i}$, where *_i_* is the iteration number.

It should be noted that the IEKF achieves good results if the measurement model is close to linear between the true state **x***_k_* and the calculated posterior intermediate state estimate after one linearization. Otherwise, the linearization error increases due to re-linearization and the IEKF fails to improve the state estimate. Thus, it's necessary to make sure that the measurement model of VTL is close to linear around the true state **x***_k_*.

In VLT GPS receiver implementation, the observation Equation *h*(·) is used to calculate the code phase errors and carrier frequency errors of each satellite channel. For easily to analyses, we suppose there is only one satellite channel, and then *h*(·) is shown in Equation (37):

(37) $$h(X_{k})=\left[\begin{array}{c} \left(\frac{(x_{r}+x_{k})-x_{s}}{\rho }\right)\cdot x_{k}+\left(\frac{(y_{r}+y_{k})-y_{s}}{\rho }\right)\cdot y_{k}+\left(\frac{({\text{z}}_{r}+{\text{z}}_{k})-{\text{z}}_{s}}{\rho }\right)\cdot z_{k}\\ \left(\frac{(x_{r}+x_{k})-x_{s}}{\rho }\right)\cdot \nu_{x,k}+\left(\frac{(y_{r}+y_{k})-y_{s}}{\rho }\right)\cdot \nu_{y,k}+\left(\frac{(z_{r}+z_{k})-{\text{z}}_{s}}{\rho }\right)\cdot \nu_{z,k} \end{array}\right]$$

where $\rho =\sqrt{{\left[(x_{r}+x_{k})-x_{s}\right]}^{2}+{\left[(y_{r}+y_{k})-y_{s}\right]}^{2}+{\left[(z_{r}+z_{k})-z_{s}\right]}^{2}}$ is the distance between GPS receiver and satellite, (*x_r_, y_r_, z_r_*) is the GPS receiver position, (*x_s_, y_s_, z_s_*) is the satellite position, *X_k_* = [*x_k_ y_k_ z_k_ v_x,k_ v_y,k_ v_z,k_*]*^T^* is the state vector, *x_k_, y_k_* and *z_k_* are the GPS receiver position error, *v_x,k_, v_y,k_* and *v_z,k_* are the GPS receiver velocity error.

It is known that the GPS satellites work on medium Earth orbit (above an altitude of 5000 km). Also, *x_k_, y_k_* and *z_k_* are usually less than 20 m. It is obvious that ρ, *x_s_, y_s_* and *z_s_* are much bigger than *x_k_, y_k_* and *z_k_* Thus, Equation (37) can be approximated to Equation (38):

(38) $$h(X_{k})\approx \left[\begin{array}{cccccc} \left(\frac{x_{r}-x_{s}}{\rho_{0}}\right) & \left(\frac{y_{r}-y_{s}}{\rho_{0}}\right) & \left(\frac{z_{r}-z_{s}}{\rho_{0}}\right) & 0 & 0 & 0\\ 0 & 0 & 0 & \left(\frac{x_{\text{r}}-x_{\text{s}}}{\rho_{0}}\right) & \left(\frac{y_{\text{r}}-y_{\text{s}}}{\rho_{0}}\right) & \left(\frac{z_{r}-z_{s}}{\rho_{0}}\right) \end{array}\right]\;\left[\begin{array}{c} x_{k}\\ y_{k}\\ \begin{array}{l} z_{k}\\ v_{x,k}\\ v_{y,k}\\ v_{z,k} \end{array} \end{array}\right]$$

where $\rho_{0}=\sqrt{{\left(x_{r}-x_{s}\right)}^{2}+{\left(y_{r}-y_{s}\right)}^{2}+{\left(z_{r}-z_{s}\right)}^{2}}$.

It can be seen that Equation (38) is a linear equation. Hence, the measurement model is close to linear function around **x***_k_*, and IEKF can achieve good results.

## 4. Positioning Test Results and Discussion

**Figure 6.**
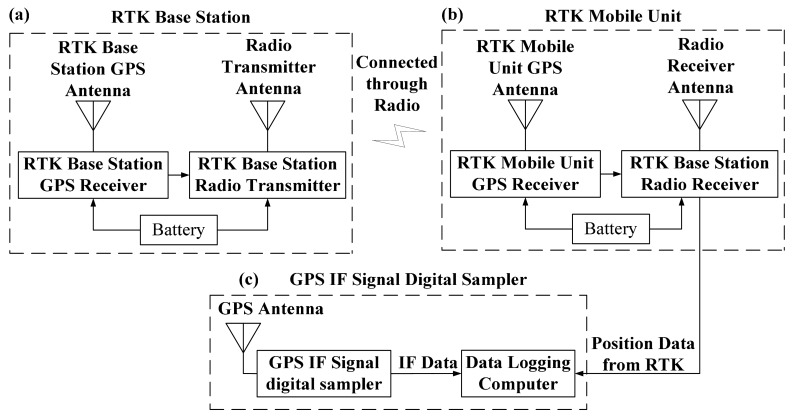
Test platform. (**a**) RTK base station; (**b**) RTK mobile unit; (**c**) GPS IF signal digital sampler.

**Figure 7.**
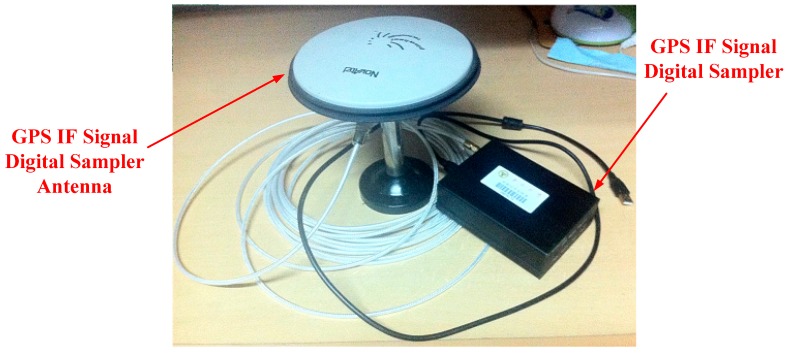
GPS IF signal digital sampler.

**Figure 8.**
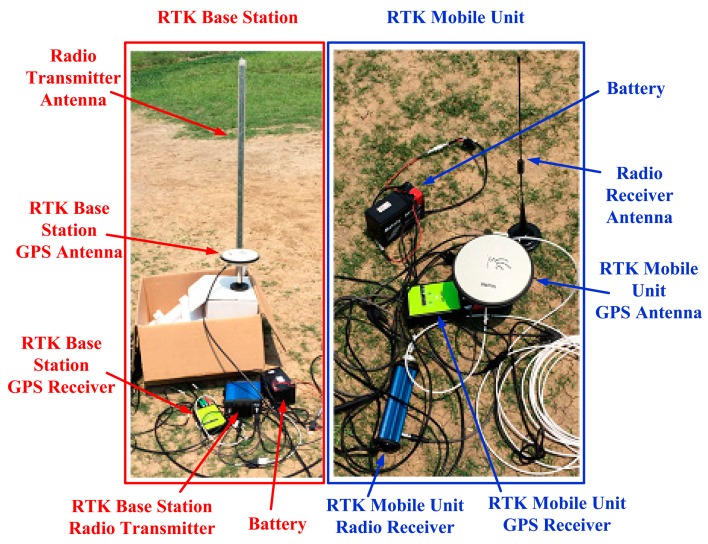
RTK system.

In this work, road tests were done to assess the performance of the proposed method. The test platform consists of a GPS IF signal digital sampler and a real-time kinematic (RTK) GPS system (Figure 6). The sampler used in this work is shown in Figure 7, and RTK system is shown in Figure 8. The characteristics of the digital sampler used in this work are listed in Table 1. The digital sampler is a digital down converter which can receive GPS signals through the GPS antenna and then convert the high frequency GPS signals down to lower frequency signals. The lower frequency signals are called intermediate frequency (IF) signals. IF signals are digitalized by analog to digital converter which is included in GPS IF signal digital sampler.

**Figure 9.**
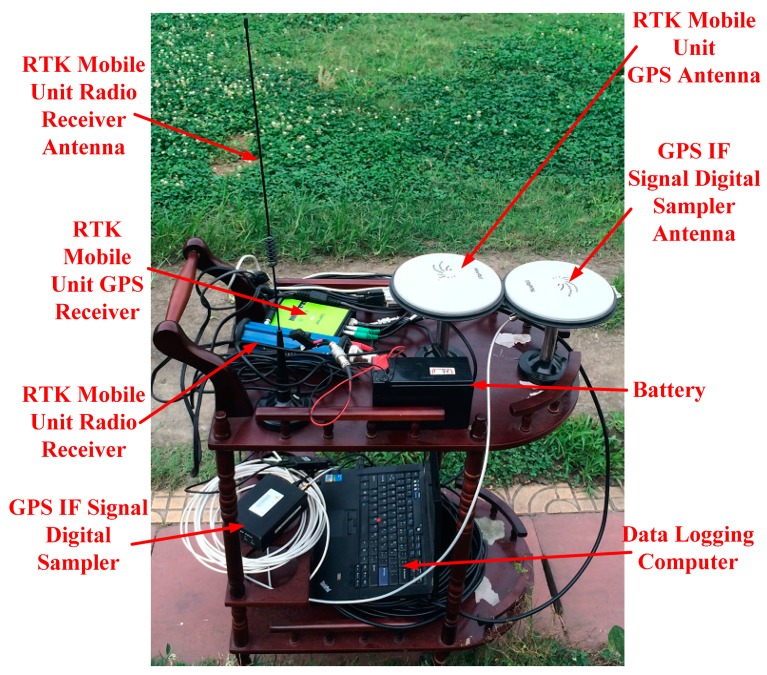
Road test.

The road test was carried out in the sports ground of Southeast University, Nanjing, China. The GPS IF signal digital sampler, GPS antenna, portable computer, and mobile unit of RTK are carried on a cart. The cart runs along the sports round one circle. The GPS IF signals are recorded by computer. In the same time, RTK mobile unit recorded the precise position of the cart (Figure 9).

**Figure 10.**
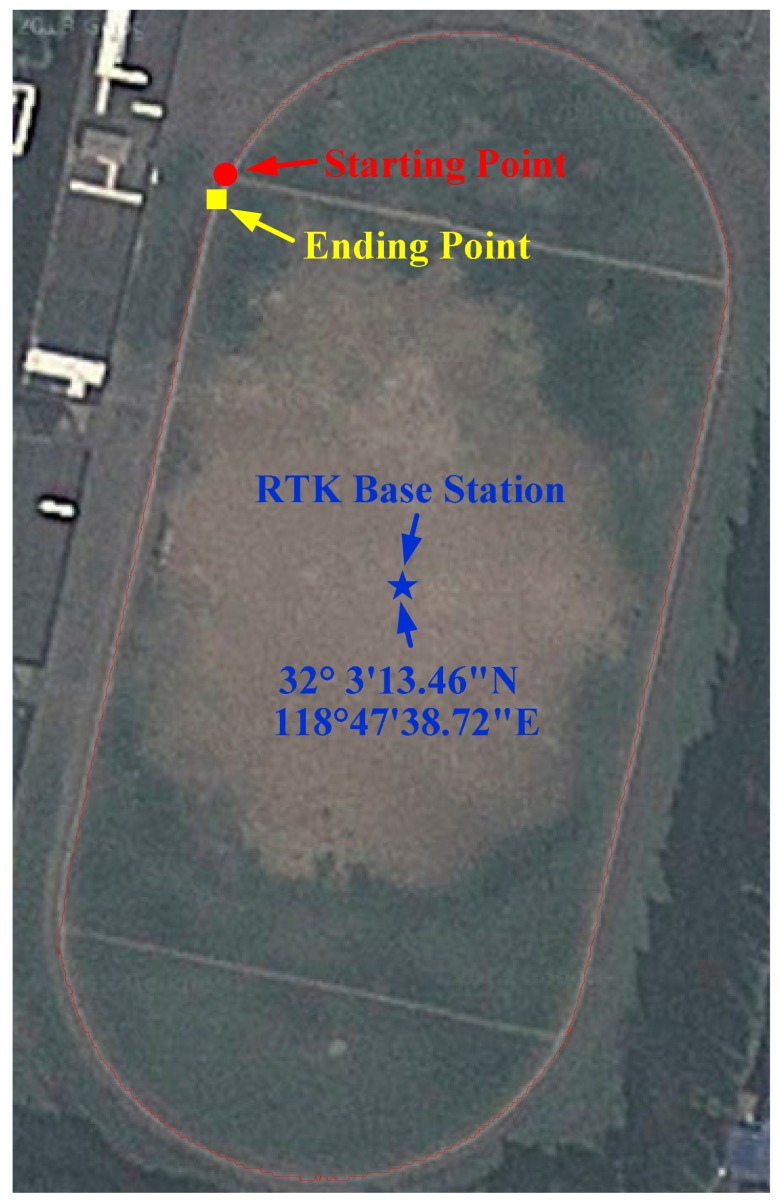
Trajectory of the cart obtained the RTK.

The DELTA-G2T RTK system is manufactured by Javad GNSS Company (San Jose, CA, USA). RTK data rate is 1 Hz. The position accuracy of RTK is 10 mm + 1 ppm. In this test, the RTK base station was placed at a known location on the sports ground (Figure 10), and the maximal distance between the GPS mobile unit and the RTK base station is less than 400 m. In this case, the RTK can provide a theoretical position accuracy of less than 10 cm. The RTK data were served as the reference in the evaluation of the VTL performance. The AIEKF implements a discretized set of differential equations described in Section 3. Differences between the estimated velocity/position and the measured ones are processed in the AIEKF, to estimate the code phase errors and carrier frequency errors. The estimated code phase errors and carrier frequency errors are used to control the numerically controlled oscillators (NCO) of code/carrier generators.

**Figure 11.**
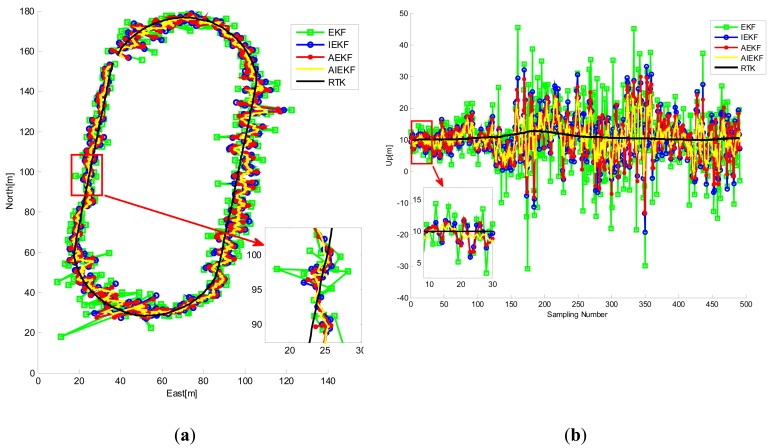
Trajectory of the road test obtained from RTK, EKF, IEKF, AEKF and AIEKF. (**a**) Longitude and latitude; (**b**) Altitude.

**Figure 12.**
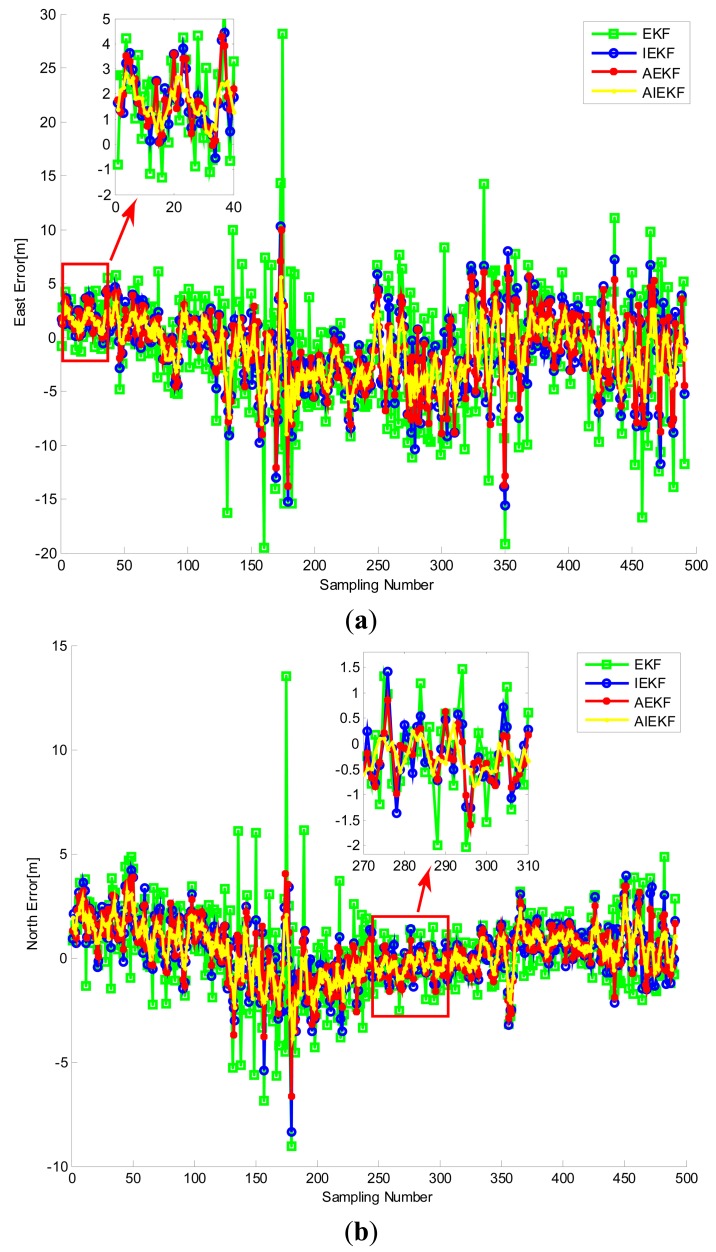

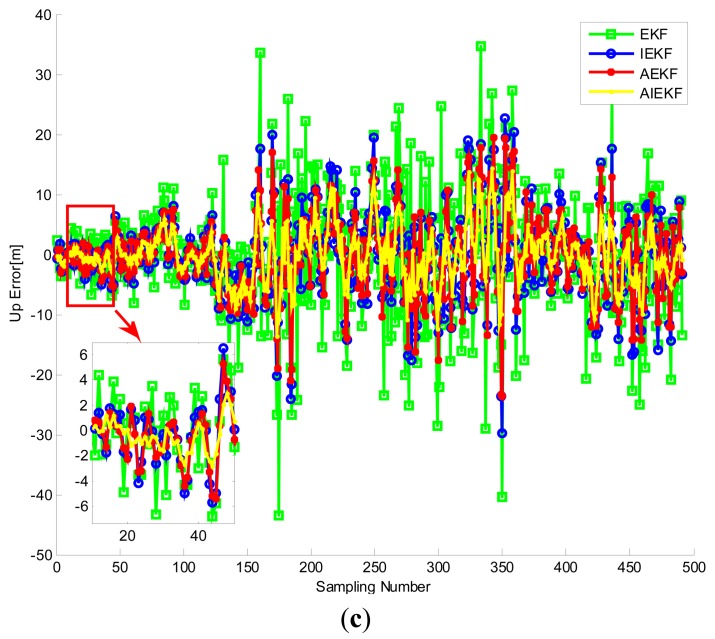
Position errors of VLL GPS receiver. (**a**) Longitude error; (**b**) Latitude error; (**c**) Altitude error.

Figure 10 shows the trajectory of the cart obtained from RTK. The road test data were processed by EKF, IEKF, AEKF and AIEKF methods respectively and the trajectories of EKF, IEKF, AEKF and AIEKF are shown in Figure 11. The position errors, code phase errors and carrier frequency errors are important performance indexes of the VTL GPS receiver. Figure 12 is the position error curves about EKF, IEKF, AEKF and AIEKF method. In Figure 12, green square line is EKF position error curve, blue circle line is IEKF position error curve, red point line is AEKF position error curve, and yellow line is AIEKF position error curve. It can be seen from Figure 12 that IEKF, AEKF and AIEKF can obtain higher accuracy on position compared with the EKF. Comparing with IEKF and AEKF method, the AIEKF method proposed in this paper can more effectively to reduce position error of VTL GPS receiver.

**Figure 13.**
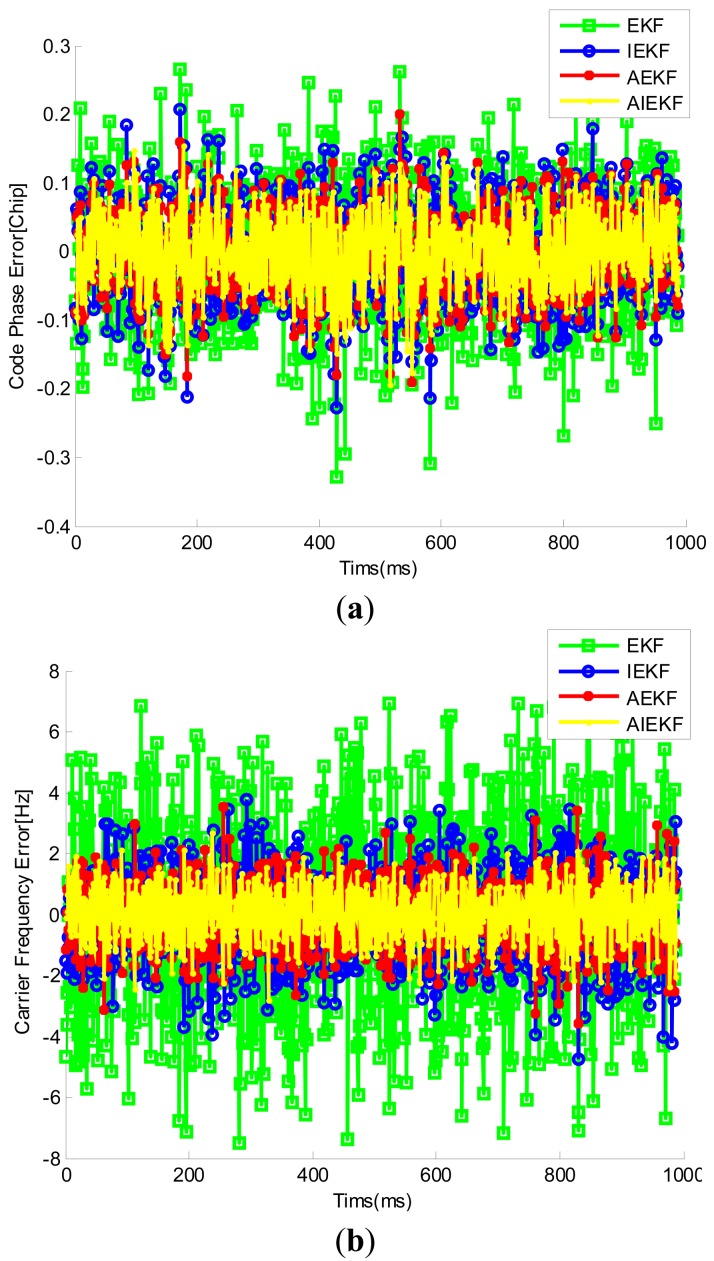
Code phase error and carrier frequency error of PRN 2 satellite. (**a**) Code phase error; (**b**) Carrier frequency error.

Table 1 shows the position RMSE values of the EKF, IEKF, AEKF, and AIEKF during the road test. From Table 1, it can be seen that, compared with EKF, IEKF and AEKF, the RMSE values of AIEKF are reduced by about 45.1%, 25.7% and 21.6% in the east direction, respectively. In Table 1, similar results can also be seen in the north and up directions. Similarly, Figure 13 shows the code phase error and carrier frequency error curves. From Table 2 we can see that, the RMSE value of code phase errors and carrier frequency errors based on AIEKF are lower compared with EKF, IEKF and AEKF. It should be noted that the code phase accuracy of tracking-loop is very important for GPS receiver. Higher code phase accuracy means higher positioning accuracy. Thus, the VTL GPS receiver based on AIEKF has better performance.

**Figure 14.**
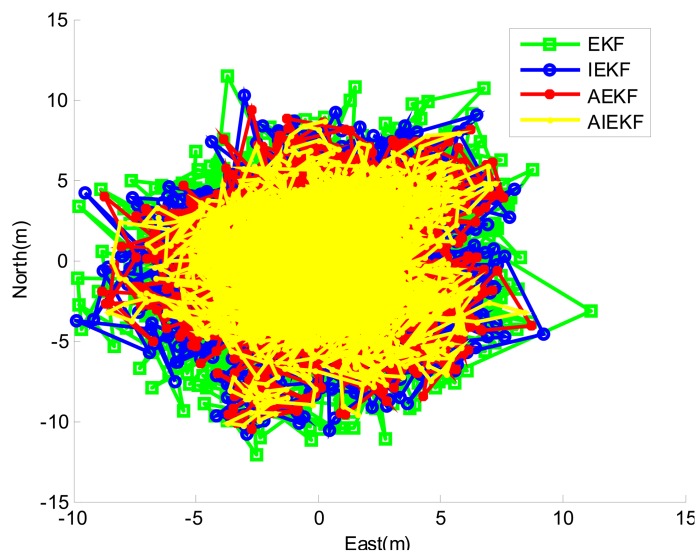
Static positioning result.

In addition to the road test, a static positioning test (1000 s) was carried out at the Baima Park square, Nanjing, China. The static positioning GPS IF signal was processed by GPS software receiver based on STL method and VTL method (based on EKF, IEKF, AEKF and AIEKF) respectively. The GPS software receiver generates a positioning result per 200 milliseconds. Figure 14 shows the static positioning results based on scalar, EKF, IEKF, AEKF and AIEKF method respectively. It can be seen from Table 3 that, compared with EKF, IEKF and AEKF, the RMSE values of AIEKF are reduced by about 14.7%, 8.1% and 4.1% in the east direction, respectively. Meanwhile, the similar results can be seen in the north and up directions.

## 5. Conclusions

This work proposed the AIEKF method for a VTL GPS software defined receiver. In this model, AIEKF is employed instead of the EKF in VTL. In AIEKF, IEKF and AEKF are used together. IEKF can reduce the truncation error of EKF by a simple iterative procedure. Furthermore, the noise statistics estimator is employed in the IEKF to combine the advantages of the AEKF and the IEKF. The experimental results show that the proposed AIEKF outperforms the EKF.

## Figures and Tables

**Table 1.** Position errors of the EKF, IEKF and AIEKF method.

**Method**	**RMSE(m)**		

	**East**	**North**	**Up**
EKF	5.10	1.98	10.05
IEKF	3.77	1.45	7.09
AEKF	3.57	1.38	6.58
AIEKF	2.80	1.17	4.56

**Table 2.** Code phase and carrier frequency errors of the EKF, IEKF and AIEKF method.

**Method**	**RMSE(Hz)**	**RMSE(Chip)**

	**Carrier Frequency Error**	**Code Phase Error**
EKF	2.73	0.088
IEKF	1.30	0.063
AEKF	1.00	0.056
AIEKF	0.84	0.051

**Table 3.** RMSE of static positioning test depending on scalar, EKF, IEKF and AIEKF method.

**Method**	**RMSE(m)**		

	**East**	**North**	**Up**
EKF	2.79	3.19	9.29
IEKF	2.59	2.97	8.51
AEKF	2.48	2.85	8.09
AIEKF	2.38	2.74	7.64

## References

[1] Kim K., Jee G.I., Im S.H. (2011). Adaptive Vector-Tracking Loop for Low-Quality GPS Signals. Int. J. Control Autom. Syst..

[2] Zhao S.H., Lu M.Q., Feng Z.M. (2011). Implementation and Performance Assessment of a Vector Tracking Method Based on a Software GPS Receiver. J. Navig..

[3] Jafarnia-Jahromi A., Lin T., Broumandan A., Nielsen J., Lachapelle G. Detection and Mitigation of Spoofing Attacks on a Vector-Based Tracking GPS Receiver.

[4] Parkinson B.W., Spilker J.J. (1996). Theory and Applications Volume I. Global Positioning System: Theory and Applications.

[5] Lashley M., Bevly D.M. Vector Delay/Frequency Lock Loop Implementation and Analysis.

[6] Petovello M.G., Lachapelle G. Comparison of vector-based software receiver implementations with application to ultra-tight GPS/INS integration.

[7] Won J.H., Eissfeller B. Effectiveness analysis of vector-tracking-loop in signal fading environment.

[8] Lashley M., Bevly D.M., Hung J.Y. (2009). Performance analysis of vector tracking algorithms for weak GPS signals in high dynamics. Sel. Top. Signal Process. IEEE J..

[9] Fang J.C., Gong X.L. (2010). Predictive iterated Kalman filter for INS/GPS integration and its application to SAR motion compensation. IEEE Trans. Instrum. Meas..

[10] Xu Y., Chen X.Y., Li Q.H. (2013). Autonomous Integrated Navigation for Indoor Robots Utilizing On-Line Iterated Extended Rauch-Tung-Striebel Smoothing. Sensors.

[11] Xu Y., Chen X.Y., Li Q.H. (2014). Adaptive Iterated Extended Kalman Filter and Its Application to Autonomous Integrated Navigation for Indoor Robot. Sci. World J..

[12] Wan E.A., van der Merwe R. The unscented Kalman filter for nonlinear estimation.

[13] Bell B.M. (1994). The iterated Kalman smoother as a Gauss-Newton method. SIAM J. Optim..

[14] Julier S.J., Uhlmann J.K. A new extension of the Kalman filter to nonlinear systems.

[15] Spilker J.J. (1994). Vector Delay Lock Loop Processing of Radiolocation Transmitter Signals.

[16] Benson D. Interference Benefits of a Vector Delay Lock Loop (VDLL) GPS Receiver.

[17] Kaplan E.D. (2005). Understanding GPS: Principles and Applications.

[18] Lashely M., Bevly D.M. Analysis of Discriminator Based Vector Tracking Algorithms.

[19] Pany T., Eissfeller B. Use of a Vector Delay Lock Loop Receiver for GNSS Signal Power Analysis in Bad Signal Conditions.

[20] So H., Lee T., Jeon S., Kim C., Kee C., Kim T., Lee S. (2010). Implementation of a Vector-Based Tracking Loop Receiver in a Pseudolite Navigation System. Sensors.

[21] Shojaie K., Ahmadi K., Shahri A.M. Effects of iteration in Kalman filters family for improvement of estimation accuracy in simultaneous localization and mapping.

